# A Novel Bayesian Method for Detection of APOBEC3-Mediated Hypermutation and Its Application to Zoonotic Transmission of Simian Foamy Viruses

**DOI:** 10.1371/journal.pcbi.1003493

**Published:** 2014-02-27

**Authors:** Frederick A. Matsen, Christopher T. Small, Khanh Soliven, Gregory A. Engel, Mostafa M. Feeroz, Xiaoxing Wang, Karen L. Craig, M. Kamrul Hasan, Michael Emerman, Maxine L. Linial, Lisa Jones-Engel

**Affiliations:** 1 Fred Hutchinson Cancer Research Center, Seattle, Washington, United States of America; 2 University of Washington, Seattle, Washington, United States of America; 3 Swedish Medical Center, Seattle, Washington, United States of America; 4 Jahangirnagar University, Savar, Dhaka, Bangladesh; University of California San Diego, United States of America

## Abstract

Simian Foamy Virus (SFV) can be transmitted from non-human primates (NHP) to humans. However, there are no documented cases of human to human transmission, and significant differences exist between infection in NHP and human hosts. The mechanism for these between-host differences is not completely understood. In this paper we develop a new Bayesian approach to the detection of APOBEC3-mediated hypermutation, and use it to compare SFV sequences from human and NHP hosts living in close proximity in Bangladesh. We find that human APOBEC3G can induce genetic changes that may prevent SFV replication in infected humans in vivo.

## Author Summary

Simian Foamy Virus (SFV) is a very common retrovirus in monkeys. When an infected monkey bites a human it can transmit the virus to the human; however, there are no documented cases of human to human transmission. There also appear to be significant differences between infection in monkey and human hosts. The reason for these differences in the two hosts is not completely understood. In this paper we show that a family of host defense enzymes called APOBEC3 may prevent replication of SFV in humans. They do this by changing the genome of the virus so that it cannot replicate. Although this same process also happens in monkeys, it appears to happen less than in humans, and the changes that the monkey APOBEC3 enzymes make are less likely to prevent the virus from replicating. We are able to make these inferences by seeing characteristic types of mutations in a collection of virus DNA sequences sampled in Bangladesh. We develop new statistical methodology to do this analysis.

## Introduction

Simian foamy viruses (SFV) comprise a subfamily of retroviruses that naturally infect all primates examined with the notable exception of humans. In non-human primates (NHP), they show strong evidence of co-evolution with their hosts [1]. Persistent infection with SFV is ubiquitous in populations of free-ranging NHP [2], [3] and is not thought to be pathogenic in the natural host. However, recent work shows increased morbidity and mortality for macaques infected with SFV and SIV (simian immunodeficiency virus) compared to those infected with SIV alone [4]. SFV has been zoonotically transmitted to humans on more independent occasions than any other simian-borne retrovirus [5], [6]. There are no documented cases of human to human SFV transmission, including between discordant couples [7], [8]. The factors underlying the apparent lack of human-to-human transmission are not well understood. However, the apparent lack of viral replication in humans is probably an important factor [7], [9]. In NHP, SFV is believed to be transmitted through saliva, primarily through biting. This conclusion is supported by studies that have shown high levels of viral RNA in the oral mucosa of NHP, indicative of replication at that site [10], [11]. The large number of NHP infected with SFV and relatively frequent zoonotic transmission allow study of the roles that viral strain variation and host immune response may play in preventing SFV from becoming an endemic human virus.

There have been no direct experimental infections of a susceptible host with SFV or any other foamy virus. However, blood transfusions from an SFV positive NHP to an SFV negative NHP have been reported [12], [13]. From these studies, a model for the events that occur after SFV infection has been proposed. Briefly, initial infection is of PBMCs. Viral DNA integrations are found in these cells, but replication is not detectable. When a latently infected PBMC migrates to the oral mucosa, an unknown process occurs that leads to infection of superficial epithelial cells, in which the virus can replicate [10], [11]. Infections are persistent, but the only cells that have been found to replicate virus are in the oral mucosa. However, almost all organs in an infected NHP contain latent proviruses at levels suggesting there are many other cell types other than PBMCs that can be latently infected.

Host-viral interactions are better understood for SIV, an NHP-borne lentivirus, than for SFV. In particular the innate immune system is known to play an important role in limiting lentiviral inter-species transmission. Host factors such as SAMHD1, tetherin, and APOBEC3 [14] are known to restrict lentiviruses, which in turn have evolved viral protein antagonists to counter these specific host factors. Cross-species transmission of lentiviruses can be limited by the specificity of these viral antagonists for the host species to which the virus has adapted [15]. The APOBEC3 family of proteins are cytidine deaminases that act on negative strand single-stranded DNA, which is created during reverse transcription. Deamination changes C to U, which then appears as G to A mutations on the positive strand [14]. The importance of APOBEC3G as a barrier to cross-species transmission of SIV has recently been highlighted by Etienne et al [16], who provide evidence that the ability of SIVcpz Vif to adapt to restrict chimpanzee APOBEC3G was more important than its ability to counter SAMHD1 with another viral gene, *vpx*.

Human APOBEC3 has also been shown to be a potent SFV restriction factor in tissue culture [17]. Some G to A mutations have also been observed in SFV sequences derived from human hosts [17]. These authors suggested that the observed mutations may have been due to APOBEC3 hypermutation, but they noted that strain-level polymorphisms, random retroviral mutations, or other processes could not be excluded as alternative explanations. Also, current methods for detecting and quantifying APOBEC3-mediated hypermutation have limited sensitivities at low rates of hypermutation. Thus, new methods are needed to resolve how APOBEC3 proteins might protect humans from zoonotic transmission of retroviruses.

APOBEC3 activity against retroviruses can be inferred via the local sequence specificity of these editing enzymes. In general, APOBEC3 activity is detectable as an overall excess of plus-strand G to A mutations, however, the various members of the APOBEC3 gene family each have their own local nucleotide context specificity [18]. Much of the work on this specificity has focused on the dinucleotide pair formed by a G and the nucleotide immediately following on the positive strand. For example, human APOBEC3G is known to induce mutation in a GG context. Thus the level of activity of a given APOBEC3 enzyme can be characterized using the counts of G to A mutations in and out of context for that enzyme. Continuing the APOBEC3G example, by comparing the number of GG dinucleotide context G to A mutations to the number of such mutations outside this context, one can detect APOBEC3G hypermutation. Similarly, hypermutation by other APOBEC3 proteins can be inferred by G to A mutations in other dinucleotide contexts.

Currently, the most popular approach, as implemented in the widely used HYPERMUT program [19], is to use a Fisher test to determine if the in context mutations statistically exceed the out of context mutations. This application of the Fisher test has three shortcomings: first, when testing the equality of two binomial distributions, the nominal p-value of the Fisher test does not correspond to the actual rejection rate under the null [20]–[23]. Indeed, by simulating under the null in parameter regimes relevant to hypermutation analysis we show that it does indeed deviate from the nominal p-value, and importantly that the level of deviation depends on the parameters and thus cannot be ameliorated by a simple global change of cut off. However, we also find that the “mid-P” variant [24] does show significantly better performance than the classical Fisher test in this respect. Second, the Fisher test does not provide an estimate of the relative probability of mutation (i.e. the effect size). Third, because the Fisher test requires a strict segregation of sites into “in context” and “out of context,” it does not provide a foundation for further generalization to incorporate subtleties such as varying “strengths” of hypermutation contexts.

In this paper, we employ a Bayesian method to detect and quantify hypermutation by estimating the relative probability, along with uncertainty estimates, of G to A mutation in a given APOBEC3-associated context versus a control context. In addition to providing a more sensitive test, the Bayesian methodology provides an integrated means to estimate effect size (i.e., hypermutation strength) and significance (to decide whether hypermutation is occurring). The risk ratio (described below) is a natural choice to report alongside the Fisher p-value for effect size estimation, as HYPERMUT does. Our approach does a better job of effect size estimation than the risk ratio for a range of parameter values spanning the data sets we have analyzed. Finally, the Bayesian approach can be directly generalized to situations such as different strengths of various hypermutation contexts.

Using this Bayesian approach, we examined the hypermutation patterns of 1097 blood proviral DNA sequences from 169 rhesus macaques, as well as 152 buccal swab RNA sequences from 30 of these animals, and compared them to the hypermutation patterns of 77 SFV proviral DNA sequences detected in blood obtained from 8 zoonotically infected humans sampled from the same geographic areas as the macaques [3], [25], [9]. The buccal swabs are important for our analysis as they represent SFV as it is actively replicating rather than latently present in blood.

For our studies of SFV variation, we have examined 1125 nucleotides of the *gag* gene [3]. This region of the genome was chosen for our studies because in FV, the *gag* sequence is the most variable of those encoding virion associated proteins [26]. This is unlike the case of orthoretroviruses, where the *env* gene is the most variable. The 1125 nucleotides were also chosen because this region contains only one short motif (PSAP) that is known to be required for FV replication. We reasoned that the relatively high variability in this region of *gag* would allow us to define viral strains. Since we had a large data set from this region of *gag* [3], [25], [9], we used these sequences to determine potential APOBEC3 mediated hypermutation of SFV.

Although we found evidence of hypermutation in SFV sequences from both humans and macaques, the relative frequency and intensity of SFV *gag* hypermutation differed significantly between macaques and humans, as did the dinucleotide contexts, suggestive of different host APOBEC3 activities. Moreover, by comparing macaque buccal swab RNA sequences to those obtained from human whole blood, we conclude that the signature of hypermutation in human host SFV sequences is not present in the viruses shed from monkey oral mucosal tissues, but likely arose after at least one round of replication in the human host. Taken together, our results indicate that human APOBEC3G is at least one mechanism that protects humans from extensive replication of some SFV strains.

## Results

### Relative probability ratio estimation to detect APOBEC3-mediated hypermutation

**Figure 1:**
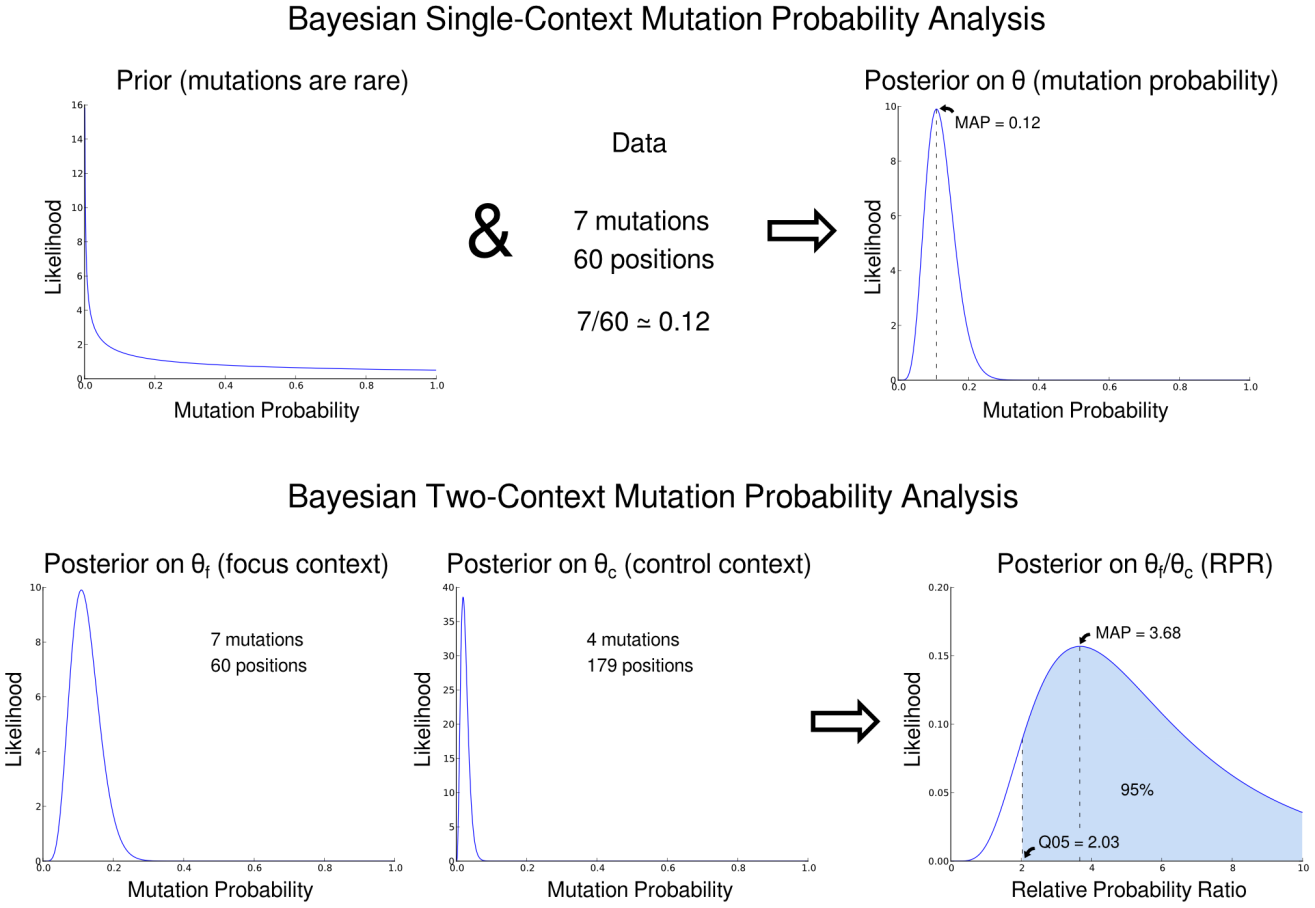
An overview of calculating the relative probability ratio (RPR). Top row: starting with a prior distribution and then adding data, we get a posterior distribution of the mutation probability given that data. Bottom row: we can do this in the *focus* context (a nucleotide context associated with hypermutation) and a *control* context (one that is not). Taking the ratio of the corresponding random variables gives the posterior on the ratio of the mutation probabilities. Using this distribution we estimate the 0.05 quantile (Q05) and the Maximum A Posteriori (MAP) estimates of the RPR.

To ameliorate the issues with applying the Fisher test described in the introduction, we developed a Bayesian approach to use the in-context versus out-of-context mutation counts to statistically identify hypermutation and quantify its strength (Figure 1). Our method uses the same data as the Fisher test to describe the ratio, with uncertainty estimates, of the probability of G to A mutation in a dinucleotide context of interest compared to the corresponding probability in a control context. We call this ratio the *relative probability ratio*. The uncertainty estimates associated with the relative probability ratio are crucial. For instance, if we see mutation in one out of four context *X* positions, and two mutations out of four context *Y* positions, then we can guess that the relative probability ratio is 1/2. However, one can make this statement with much higher certainty if we have 1000 out of 4000 *X* context mutations and 2000 out of 4000 *Y* context mutations.

This notion of an estimate with uncertainty can be formalized using Bayesian statistics as the *posterior distribution* of a model parameter given the data. In our setting, the model parameter of interest is the relative probability of G to A mutation in a dinucleotide context associated with a particular APOBEC activity, the *focus context*, to the probability of the same mutation elsewhere, the *control context*. This relative probability will be simply quantified as the ratio of the probabilities that we will call the *relative probability ratio*.

We use two summaries of the posterior distribution of the relative probability ratio. The first is the location of the 0.05 quantile, which we abbreviate Q05. Q05 signifies the level for which, with posterior probability 0.95, the analysis predicts that the true relative probability ratio is greater than or equal to Q05. In casual terms, if Q05 is equal to 2, then we are 95% sure mutations in the focus context occur at least twice as frequently as those in the control context. We call the sequence as hypermutated in a given context when the corresponding Q05 value of the posterior distribution for the probability ratio exceeds 1.

The other summary used is the Maximum A Posteriori (MAP) value for the relative probability. The MAP is the most likely value, or mode, of the posterior distribution. As such it represents our best estimate of the relative probability ratio. It is important to note that the MAP of this ratio, the object of interest to us, is not the same as the ratio of the MAP numerator and MAP denominator. The difference between the two is especially apparent when the distributions on the numerator and denominator have substantial skew, as is often the case in our setting where the bulk of the probability can be on one side of the MAP value for each distribution. Indeed, the difference between the MAP of the ratio of two Beta-distributed random variables and the corresponding ratio of the MAP values can get arbitrarily large (Figure S1).

Note that we will be testing “overlapping” contexts such as GG and GR (G followed by a G or an A). When GR is preferred over GG, for example, this means that the combination of mutation in the GG and GA contexts was more significant than considering GG sites alone. For each sequence identified as hypermutated in more than one context, the context with the highest Q05 value was identified as the *call pattern*. The call pattern thus represents the context in which evidence of hypermutation is strongest.

Validations were carried out on mutation counts simulated from a range of relative probability ratios and background mutation probabilities (see Materials and Methods). Ideally, according to the definition of the p-value, one would get a uniform distribution of p-values under the null. Although it is not possible to get an exactly uniform distribution under the null in a discrete setting such as the Fisher test, it is desirable to have this distribution as close to uniform as possible (e.g., [24]). Under a variety of simulation conditions, we find that the classical Fisher test is far from having a uniform distribution under the null in that the observed p-value is consistently smaller than the nominal p-value. Thus, we confirm in this parameter regime the observations of others that the Fisher test is consistently “conservative.” These simulations showed that our method is more sensitive than the Fisher exact test (Table 1), and that the sensitivity of the classical Fisher test cannot be improved by a simple predetermined change of cutoff (Supplementary Figures S2 & S3). We note that our method is slightly “liberal” for some parameter regimes (in particular for testing the range between 0.05 and 0.1) and conservative for others.

**Table 1:** The positive rate of Fisher test (before/), mid-P test (between/), and our methodology (after/) under various simulated relative probability ratios.

		Simulated relative probability				
	Cutoff	1	2	4	8	16
	0.0125	0.001/0.004/0.025	0.008/0.023/0.072	0.097/0.168/0.22	0.472/0.575/0.616	0.956/0.968/0.976
	0.025	0.004/0.004/0.03	0.023/0.029/0.094	0.169/0.205/0.315	0.575/0.676/0.725	0.97/0.984/0.989
	0.05	0.009/0.031/0.035	0.047/0.096/0.119	0.274/0.339/0.389	0.718/0.756/0.81	0.989/0.992/0.994
	0.1	0.031/0.062/0.118	0.096/0.183/0.274	0.339/0.475/0.553	0.756/0.862/0.898	0.992/0.997/1

The rows show a variety of different statistical cutoffs, and columns show a variety of relative probability ratios. The rejection frequency of our method is closer to the cutoff under the null hypothesis, and is more frequently able to find a difference when one exists. These simulations were based on simulated sequences of 1200 bp, with 1/16 of sequence positions in the focus context, and 3/16 in a control context, and with a background (control context) G to A mutation probability of 0.008.

**Figure 2:**
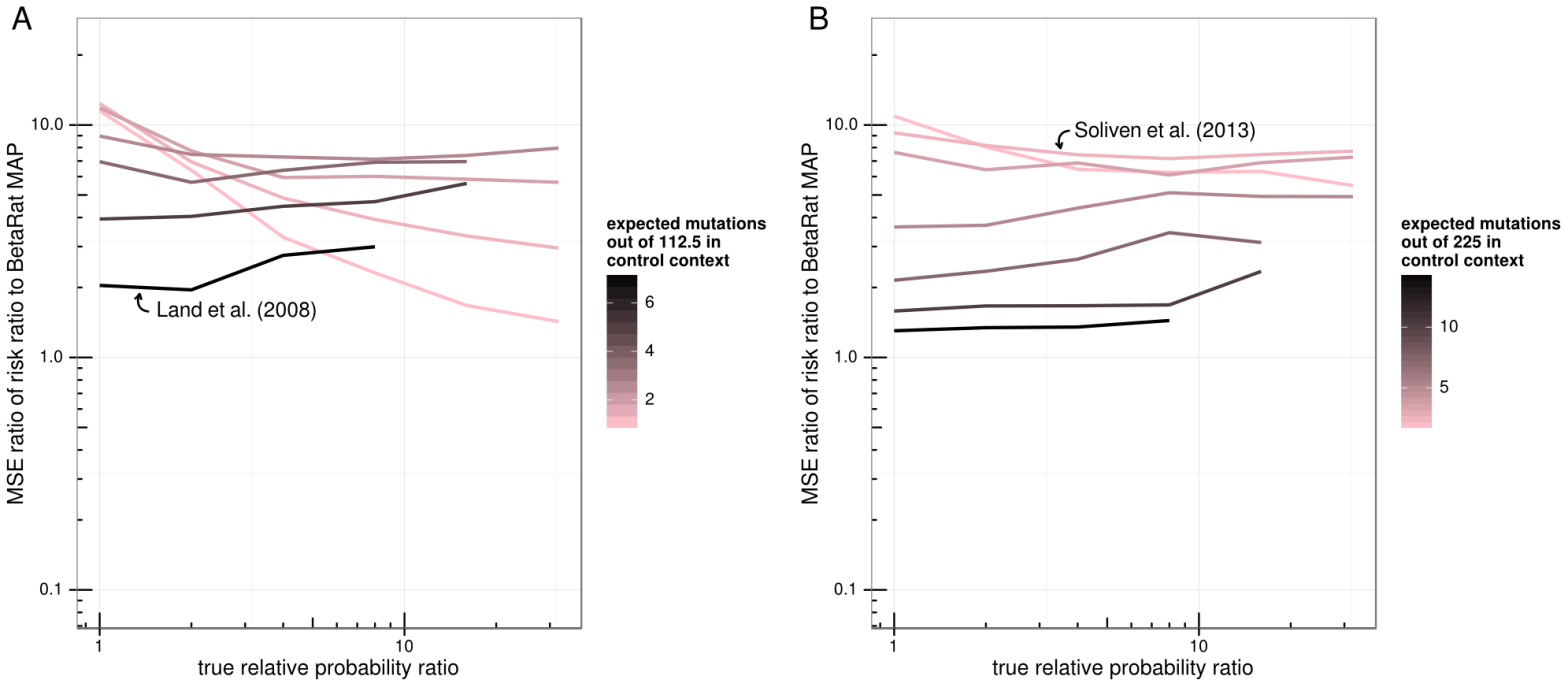
Comparison of MAP to mid-P and RR effect size estimates based on mutation count simulations of 600 bp (A) and 1200 bp (B) length sequences. The ratio of the mean squared error (MSE) of the RR estimate to that of the MAP estimator is plotted for each simulation parameter set. Points are grouped into lines and colored by control context mutation probability. The x-axis shows the relative probability ratio used for simulation. MSE ratio values greater than one indicate parameter regimes where MAP estimator does better than the RR or the mid-P estimator. Note that because RR isn't necessarily well-defined when one of the counts is zero, pseudocounts were added (see Materials and Methods). Arrows label simulations in the parameter regime of the indicated study.

Additionally, the simulations allowed us to directly compare our MAP estimates to the true relative probability ratios used to generate the simulated data. Typically researchers have calculated effect size (hypermutation strength) by the risk ratio (RR, also known as relative risk), as is done on the HYPERMUT web site (see Materials and Methods). For most of the parameter domain, MAP estimates were consistently closer to the relative probability ratios used for simulation than were the RR estimates in terms of mean squared error (Figure 2). The simulation parameter regime for this figure was chosen to span the range observed in the SFV and HIV sequences used in this study.

The “mid-P” variant of the Fisher exact test (reviewed in [24]) splits the probability of the observed contingency table in half, and assigns one half of the probability to the “more extreme table” category and half to the “less extreme table” category. This variant performed significantly better than the classical Fisher test in generating an appropriate p-value distribution (Supplementary Figures S2 & S3). For the simulations performed in this paper, this effectively corrected the issues of p-value cutoff observed with the classical Fisher test. However, the current methodology for hypermutation detection uses the classical Fisher test, rather than the mid-P version. Furthermore, in terms of the Receiver Operating Characteristic (ROC) curve to judge the true positive rate as parameterized by the false positive rate, the Bayesian approach performs slightly better than the mid-P approach (Figure S4).

We also validated our method using sequence data from an in vitro study by Refsland et al. [27], which involved knocking out members of the APOBEC3 family from human cell lines and measuring the consequent levels of hypermutation. On the Refsland data set, our methodology detected significantly more positives when the corresponding APOBEC was present, and the two tests had equal false positive rates when it was not. (Table S1). Using simulations based on the Refsland sequences, with no context-specificity to their mutations (see Materials and Methods), we see that the median positive probability for our method is below the expected 5% (Table S2).

In addition, we validated our method by applying it to sequence data from a study by Land et al. [28] that found a significant correlation between CD4 count and presence of strongly hypermutated HIV virus. We performed a similar analysis as in the original paper but with a slightly different bioinformatics pipeline, (see Materials and Methods) and did not see a significant effect when applying the Mann-Whitney test to compare CD4 counts between hypermutation positive and negative calls made by either the Fisher test or our approach. However, when we added the requirement that sequences considered positive for hypermutation by Q05 also have a large effect size as measured by MAP (in the top 25%) we did find a significant elevation in CD4 count compared to the rest of the sequences (p = 0.026). However, we did not see a significant effect when taking sequences that were positive according to mid-P and in the top 25% of effect size according to risk ratio (p = 0.31). Additionally, when restricting to the sequences found to be hypermutated, we find a much more significant nonparametric positive correlation between effect size and CD4 count using our method (Kendall tau p = 0.0026) than using mid-P together with the risk ratio (p = 0.060). These findings emphasize the importance of accurate effect size estimation, which forms an important part of our analyses of SFV sequences below.

Thus, a Bayesian framework to directly estimate the relative probability of mutation in or out of a given APOBEC3 context avoids problems associated with applying the Fisher test and provides a more accurate means for quantifying the level of hypermutation than previously described. The corresponding code is already publicly available (http://github.com/fhcrc/hyperfreq; see Materials and Methods for details) and will be made available as a web tool in the near future.

### More human host SFV sequences are hypermutated, and to a higher degree than macaque host SFV sequences

In order to investigate whether APOBEC3 activities alter SFV in macaques and/or humans infected with the virus, and to compare the levels of APOBEC3 activities in humans and macaques, we analyzed SFV *gag* sequences from a diverse collection of human blood samples as well as macaque blood and buccal samples collected across multiple urban and forested locations in Bangladesh [3], [25], [9]. Overall, 50 out of 77 (∼65%) human host SFV sequences obtained were found to be affected by hypermutation (Table 2). SFV from all but one of the 8 humans showed evidence of APOBEC3G hypermutation in at least one sequence. The exception was one individual (BGH150), whose 6 SFV clones showed no evidence of G to A hypermutation in any context. We note that the BGH150 sequences were similar to those detected in the macaques from the same region, indicating that the sequences were not amplified from contaminating plasmid. In two of our human subjects, both of whom were infected by more than one SFV strain, we observed hypermutation in clones corresponding to only one of the viral strains. Although buccal swabs were taken from the humans sampled as part of this study, none of these tested positive for SFV.

**Table 2:** Hypermutation activity by strain, presented on both a sequence by sequence and host by host basis.

Species	Strain	# positive seqs	Seq count	# positive hosts	Host count
human	bormi1	12	17	2	2
human	bormi2	21	31	4	4
human	charmaguria	0	5	0	1
human	dhamrai	4	4	1	1
human	dokhola	2	2	1	1
human	karamjal	0	7	0	2
monkey	bormi1	7	117	3	23
monkey	bormi2	1	102	1	24
monkey	charmaguria	5	157	5	27
monkey	dhamrai	26	274	15	51
monkey	dokhola	7	138	5	29
monkey	karamjal	1	66	1	10

These counts are only for core strains. Additionally, since both monkeys and humans are frequently infected with more than one strain, the host counts for a given strain represent the total number of animals infected with that strain, even if infected with other strains as well.

**Figure 3:**
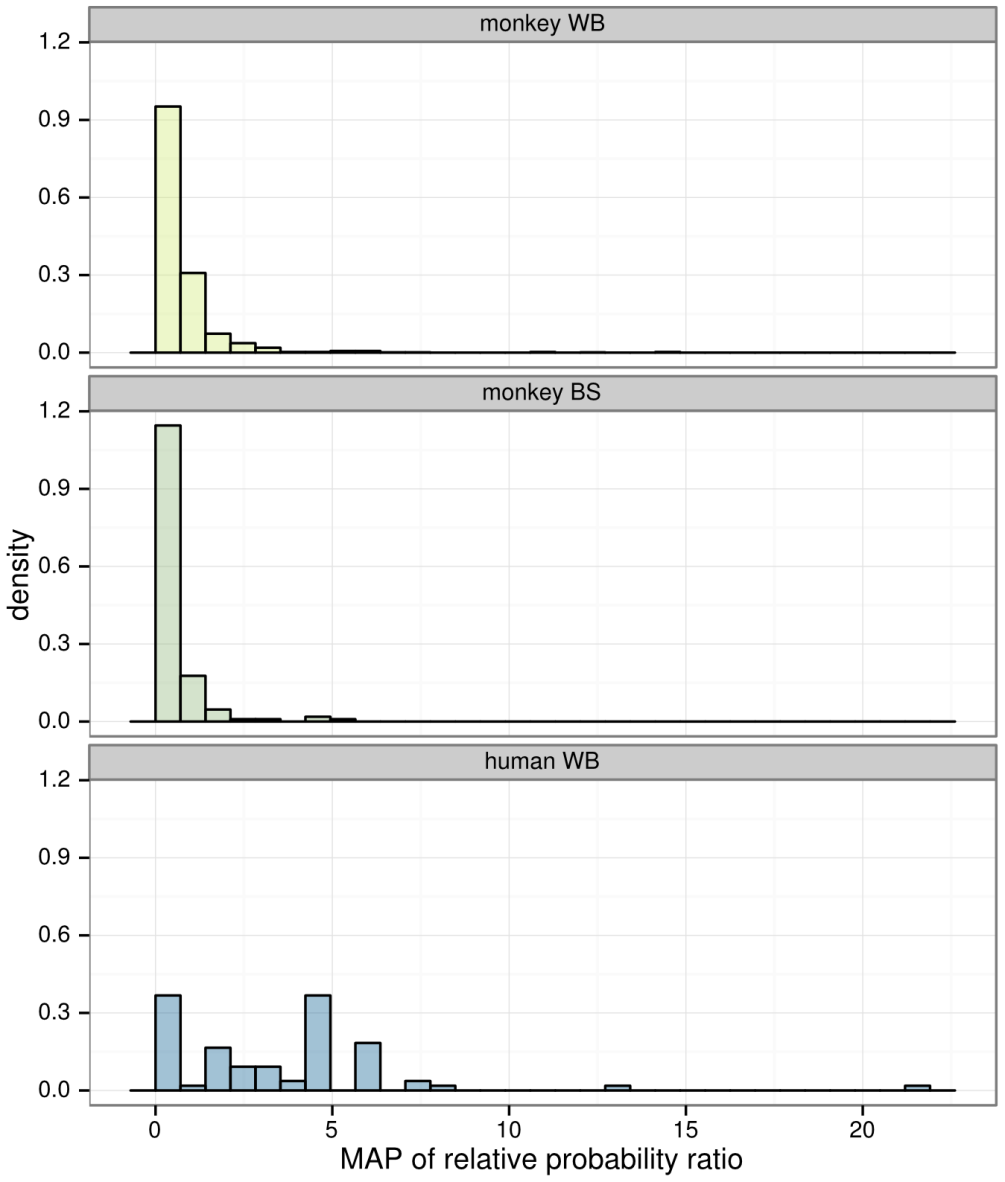
Histogram of the Maximum A Posteriori (MAP) of relative probability ratios for all sequences in the study. The distribution of the 8 human whole blood (WB) samples is to the right (towards larger values) compared to the 169 WB and 30 buccal swab (BS) samples from monkeys. The maximum of the relative probability ratio density for monkey WB samples is about 4, but the y axis of this figure was truncated for clarity.

**Figure 4:**
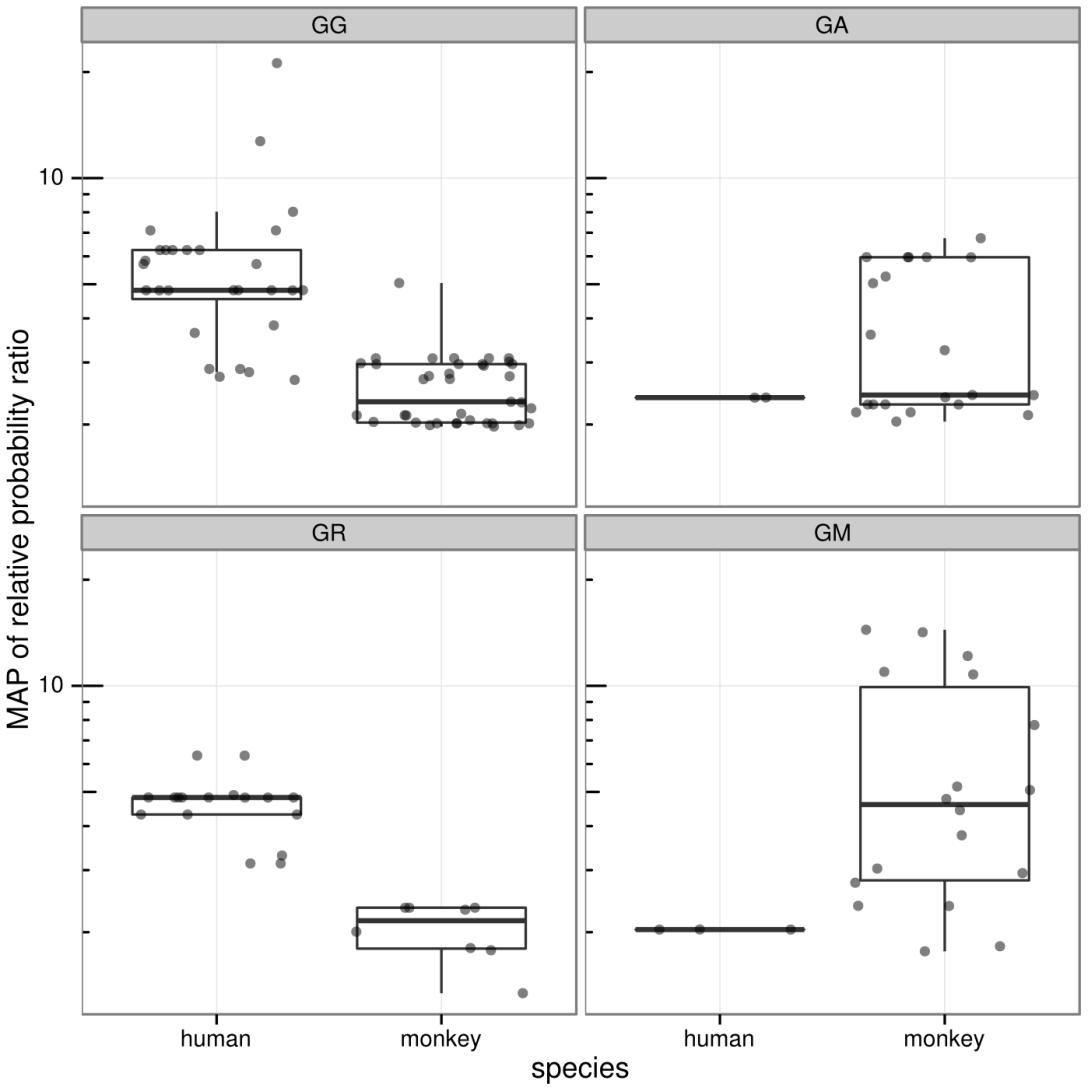
Viral sequences show distinct hypermutation profiles in the two host species, congruent with activity observed in other studies. Box and whisker plots on the same data are overlaid, where the thick horizontal bar shows the median value of the observations and the rectangle spans the first and third quartiles; points are randomly “jittered” horizontally within a species to avoid superimposed points. Panels labeled by target context using IUPAC degenerate notation, thus “R” designates A or G, and “M” designates A or C.

In contrast, only 82 out of 1097 (∼8.1%) of SFV sequences from monkey blood were found to be hypermutated, and only 42 of the 169 monkeys sampled had at least one hypermutation-positive sequence. Hypermutation was more prevalent in human blood sequences than monkey blood sequences (Fisher p = 1.3×10^−32^). Defining a sample to be hypermutated if at least one sequence obtained from the sample was hypermutated, hypermutation was more prevalent in human blood samples compared to monkey blood samples (Fisher p = 1.7×10^−4^). Additionally, the distribution of relative probability ratio across all sequences, irrespective of inferred hypermutation status, was higher for human host SFV sequences than for monkey host sequences (Figure 3). Furthermore, sequences marked as hypermutated showed a higher relative probability ratio of hypermutation in human blood than in monkey blood (Bonferroni-corrected Wilcoxon p = 1.9×10^−6^). Different context patterns were observed between human and monkey sequences (Figure 4).

**Figure 5:**
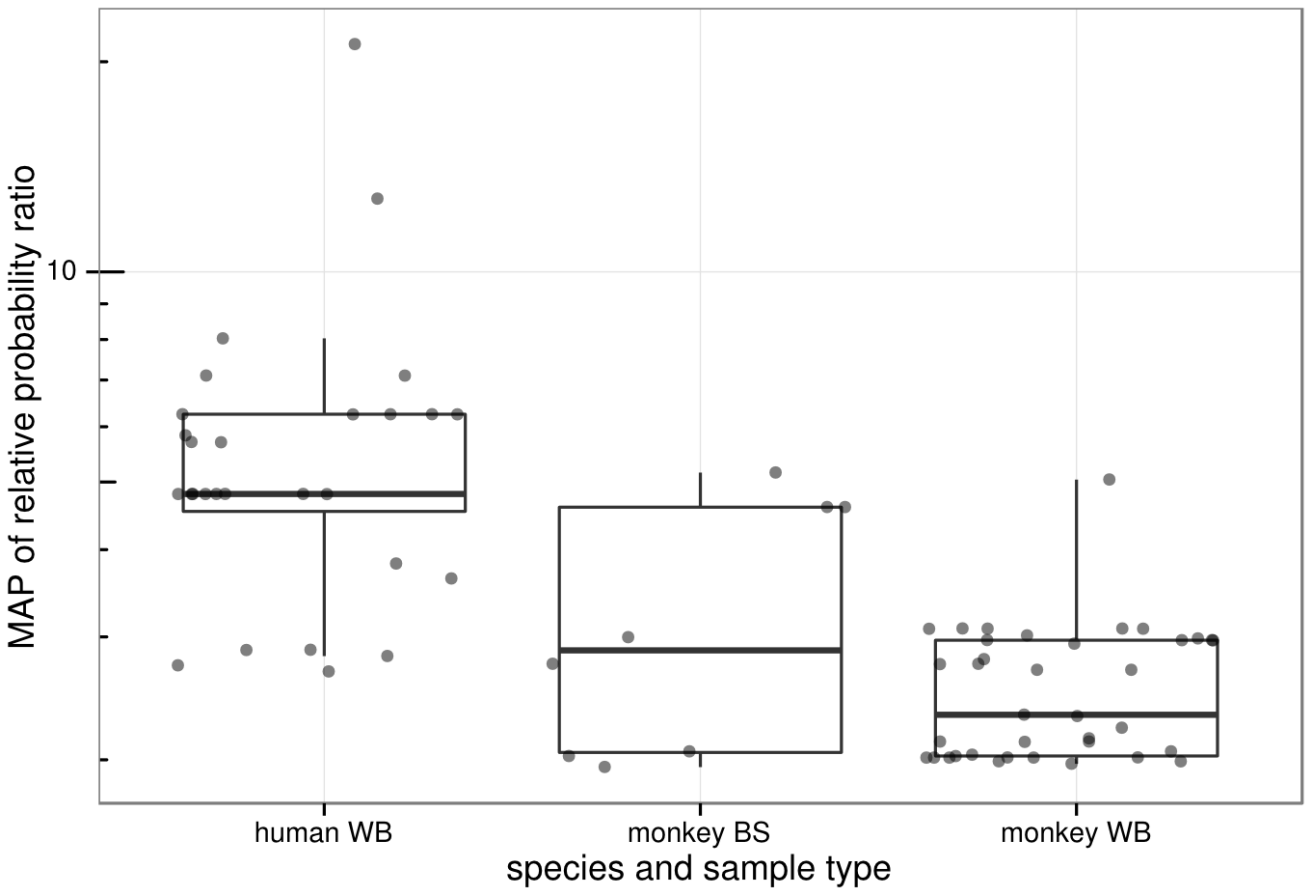
Comparison of GG context hypermutation signal in human blood, monkey blood and monkey buccal sequences. Box and whisker plots are shown as in Figure 3. The strongest hypermutation signal is observed in the human sequences.

Of the 152 sequences obtained from the 30 macaque buccal swab samples, only 8 – from 5 samples – were found to be hypermutated. Thus, hypermutation was also more prevalent in human blood sequences than monkey buccal sequences (Fisher p = 2.3×10^−22^). Similarly, more human blood samples had evidence of some hypermutation than monkey buccal samples (Fisher p = 4.3×10^−4^). Furthermore, the MAP relative probability ratios of monkey buccal sequences were significantly lower than those of the GG positive human blood sequences (Figure 5; Bonferroni-corrected Wilcoxon p = 0.023). While the frequency of hypermutation observed in monkey blood samples is higher than that of monkey buccal samples, no statistical significance was found for this relationship.

Thus, overall, with a high degree of statistical significance, more human host SFV sequences were found to be hypermutated than monkey host SFV sequences, and human host SFV sequences had a higher level of hypermutation than the SFV sequences from the macaque host.

### Hypermutation dinucleotide context is significantly different between human host and macaque host SFV

Hypermutation of human host sequences in these data was most frequently associated with the GG and GR (i.e. GG or GA) dinucleotide contexts (45 out of 50 sequences; 90%), consistent with APOBEC3G activity as well as combined APOBEC3G and APOBEC3F activity [27]. In contrast, monkeys exhibited a significant amount of GA and GM (i.e. GA or GC) context hypermutation (37 out of 82 sequences; 45%). GM context hypermutation was also observed in a study that examined hypermutation of the XMRV retrovirus in macaques [29]. Overall, hypermutation in human host sequences was more likely to be called in GG and GR contexts than for monkey host sequences (Fisher p = 1.3×10^−5^). Furthermore, human blood SFV sequences identified as hypermutated in GG and GR contexts exhibited higher MAP relative probabilities than macaque blood SFV sequences (Bonferroni-corrected Wilcoxon p = 4.8×10^−8^ and p = 3.7×10^−4^, respectively for the two contexts), corresponding to stronger action of APOBEC3G. The GM context, characteristic of macaque APOBEC3DE hypermutation [29], showed elevated levels in SFV from macaque samples (Figure 4). While the 8 monkey buccal sequences (out of 152) marked as hypermutated all exhibited the strongest hypermutation signal in a GG context, as mentioned above, the strength and abundance of this hypermutation signal was significantly lower in monkey buccal samples than human blood samples.

### There are more stop codons in human host SFV sequences than in monkey host SFV sequences

Of the 77 human blood sequences, 36 (46.8%) contained stop codons within the coding region when the sequences were translated. These stop codons were “in-frame” in that they were the result of a point mutation rather than insertion or deletion and a consequent frame shift. In contrast, only 63 of the 1097 (5.7%) monkey blood sequences had such stop codons. Thus, such stop codons are more likely in blood samples from humans than those from monkeys irrespective of whether the entire sequences were called hypermutated by any test (Fisher p = 2.2×10^−16^). When considering only sequences called hypermutation positive, this statistical relationship held (Fisher p = 6.5×10^−13^). The same was true when looking at only GG context positive sequences (Fisher p = 1.0×10^−12^). Stop codons were correlated with presence of hypermutation activity in humans: all human sequences with stop codons were classified as hypermutated, and only 15 human host sequences called hypermutation positive lacked stop codons. Thus we find that the number of stop codons in sequences from human host blood samples is statistically significantly higher than in monkey host blood sequences.

6 of the 152 (3.9%) monkey buccal swab sequences had in-frame stop codons. Thus, stop codons are also significantly more prevalent in human blood sequences than they are in monkey buccal sequences (Fisher p = 1.1×10^−14^). While the empirical frequency of stop codons is higher in monkey blood samples than in buccal samples, this relationship was not found to be statistically significant.

**Figure 6:**
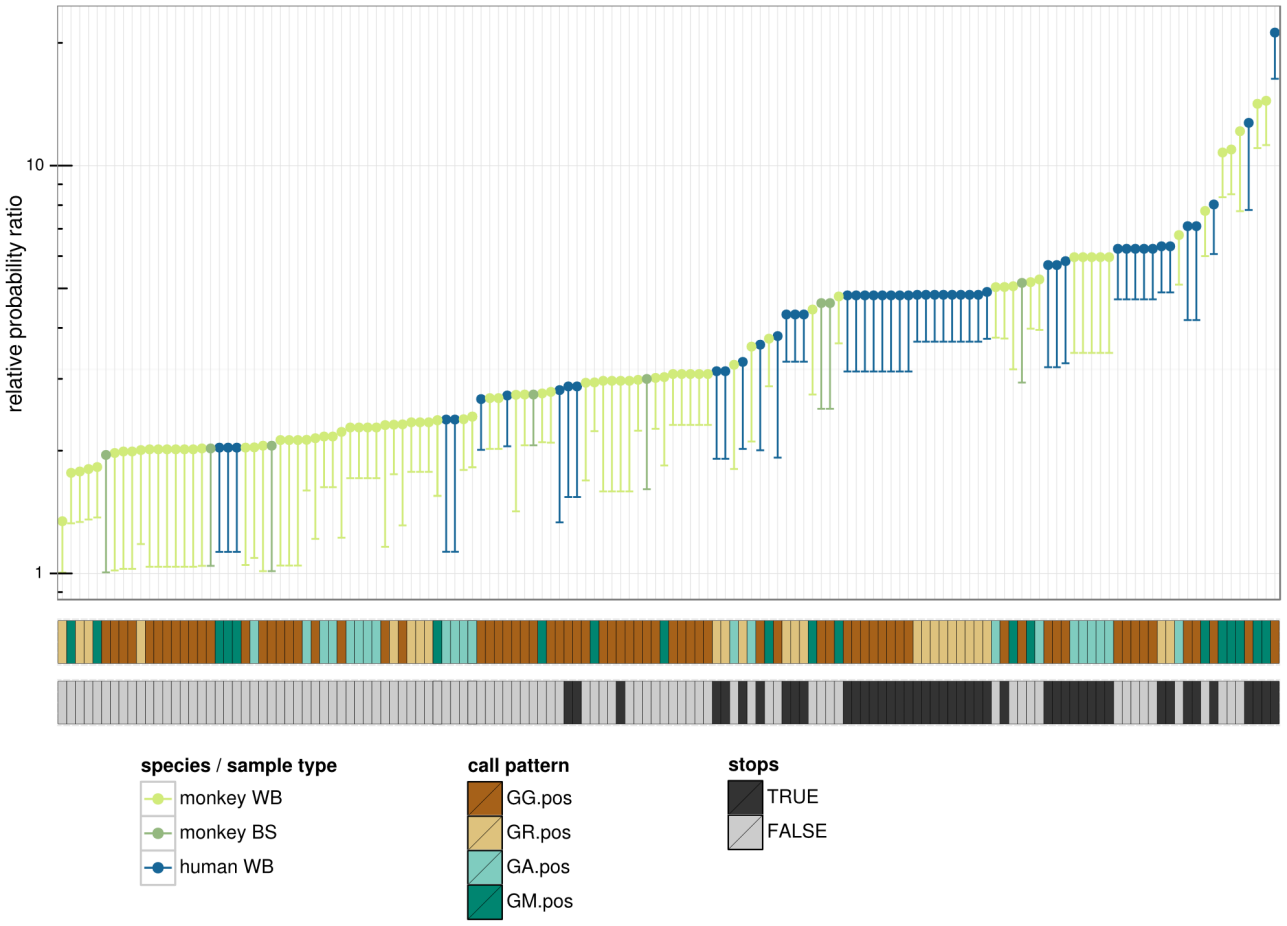
Overview of sequences found to be hypermutated. Every sequence found to be hypermutated in our data set has a column (51 of 77 human sequences, and 105 of 1097 monkey blood sequences and 8 of 152 monkey buccal sequences). The top plot represents hypermutation intensity, where the dot shows the Maximum A Posteori (MAP) value for the relative probability ratio and the lower limit of the line shows the 0.05 quantile. Sequences colored by species and sample type (whole blood (WB) or buccal swab (BS)). The call pattern is the context in which the strongest dinucleotide hypermutation signal was found (using IUPAC degenerate nucleotide notation). “Stops” signifies the presence of in frame stop codons.

Overall, by applying Bayesian analysis we show that hypermutation is statistically more prevalent, stronger and in distinct dinucleotide contexts in the human host sequences, and correlates with the presence of stop codons in a coding region for *gag* that would preclude virus replication (Figure 6).

## Discussion

### Methodology for detection and quantification of hypermutation

We have developed Bayesian methodology to test for and quantify the strength of hypermutation. Our motivation for doing so was to quantify the relative probability of mutation in various nucleotide contexts. This Bayesian method tidily formalizes this idea as estimation, with uncertainty, of the ratio of probability of mutation in two contexts as a ratio of beta-distributed random variables. This enables a unified approach to significance testing (hypermutation detection) and effect size (hypermutation strength) estimation. We show that the Bayesian effect size estimate performs better than the classically-used risk ratio (henceforth RR) over a range of parameter values (Figure 2). Additionally, it is recognized in the statistics community that the Fisher test is only appropriate when the “marginals”, i.e. the row (in this study the number of mutants versus not) and column (in this study the number of sites in dinucleotide context versus not) sums, are fixed in advance [21]. This is not the case for hypermutation detection. A number of statistical papers have highlighted problems with applying the Fisher test when this assumption is violated [20]–[23]. For example, by direct enumeration of tables, D'Agostino et al. [20] have shown that the Fisher test does not produce appropriate p-values when testing the equality of two binomial distributions. In our simulated data we also find that the classical Fisher test is less sensitive than our method (Tables 1 and S1), and that this lack of sensitivity cannot be easily remedied by considering alternate globally-applied cut-offs (Figures S2 & S3). However, the “mid-P” variant of the Fisher test does generate a null distribution that is significantly closer to the uniform than the classical Fisher test and consequently is more sensitive. This variant should be preferred to the classical Fisher test when sensitive detection of hypermutation is desired using a Fisher-type test.

Others have proposed alternate means of investigating hypermutation. One approach is to test ratios derived from k-mer motif frequencies in sequences with a Hotelling T^2^ test [30]. This method has the advantage of not needing to have every sequence paired with a putatively non-hypermutated sequence, however, it requires long sequences to get sufficient power (in that paper they used whole HIV genomes). Another group [31] has made a software package to investigate potential hypermutation using plots, but did not formalize a statistical methodology.

Using validation and an application to real data, we have shown that the Bayesian framework is an appropriate way to analyze hypermutation-by-context data and that it avoids issues associated with applying the Fisher exact test in this setting for significance testing. We also show that the effect size estimates, which follow naturally from our framework, are more accurate than the standard risk ratio estimator.

A further advantage of the Bayesian framework proposed here is that it can incorporate diverse sources of information as well as uncertainty of “hidden” variables in a principled way. We will take advantage of this feature in future work. Specifically, our next step will be to account for a variety of “strengths” of k-mer context specificities. We are motivated by observations that some contexts are more strongly associated with hypermutation than others [32], [18], [33]. Thus it is not possible to strictly segregate motifs into “hypermutation associated” versus not, making it impossible to apply tests such as the Fisher exact test.

This flexibility comes at the cost of some non-trivial computation. Indeed, although we are able to employ a closed form expression for the probability density function in a ratio of Beta distributions, this expression involves hypergeometric functions that take work to evaluate beyond standard implementations of these functions. This is in contrast with the FET and the RR estimators, which are easily implemented and computationally efficient.

The code used to evaluate sequences for hypermutation using our posterior estimation framework is available at http://github.com/fhcrc/hyperfreq. This program, as well as the routines to perform clustering to find representative non-hypermutated sequences, will be made into a more user-friendly form released within the next year and linked to from the same *hyperfreq* website.

### Hypermutation in Simian Foamy Virus

Using this methodology we found that hypermutation in SFV latent proviral sequences from zoonotically infected humans is common, strong, and primarily in the GG dinucleotide context with some in GA and GR (i.e. GG and GA combined). This corresponds primarily to APOBEC3G activity, perhaps combined with activity of another APOBEC3. In contrast, the hypermutation signal observed in macaques is rare, generally much weaker, and in a distinct set of dinucleotide contexts. A relatively small number of these sequences exhibit very strong GM (i.e. G followed by A or C) and GA context hypermutation, suggestive of rhesus macaque APOBEC3DE activity [29].

By quantifying the strength, frequency, and context specificity of APOBEC3 acting on SFV, we show that it is likely an important restriction factor that acts *in vivo* to limit replication of some SFV strains in the human host (Figure 6). This is true not only when comparing hypermutation levels between proviruses present in human blood and monkey blood, but also when comparing SFV sequences present in human blood and monkey buccal swabs. This is important, as oral mucosal tissues are the apparent source of infectious virus. APOBEC3G-mediated inhibition of replication in humans could explain the lack of human to human transmission of these strains.

The differences in hypermutation context and strength suggest that the observed hypermutation in human host sequences could not have originated in macaques prior to transmission, and must instead be occurring within human hosts. Other researchers have shown human APOBEC3 to be a potent SFV restriction factor *in vitro* [17]. These researchers also observed G to A mutations in SFV sequences derived from four bushmeat hunters from Southern Cameroon [17]. These individuals were persistently infected with gorilla SFV from 10 to 30 year old bites, and viral loads in PBMCs were described as being low. Several G to A mutations were observed, some of which were in GG and GA contexts, which may be explained by APOBEC3G or APOBEC3F activity that targeted the viruses. However, the authors of that study did not take a statistical approach and stated that they could not rule out alternate causes for the observed mutations. Thus the present study is the first to clearly show human APOBEC3 activity against SFV *in vivo*.

There are conflicting data on whether or not there is an SFV viral antagonist to APOBEC3 analogous to lentiviral Vif. While some researchers [34]–[36] report that the nonstructural protein Bet can counteract APOBEC3 activity, others [17] have not been able to detect a difference between restriction of wild-type viruses and viruses lacking Bet. However, it is possible that viruses can evade APOBEC3 using other mechanisms. For example, murine leukemia virus does this via modification of the Gag protein rather than through a specific viral antagonist [37], [38]. In either case, our data support a model where some strains of SFV are sensitive to inactivation by human APOBEC3G.

APOBEC3 enzymes work on ssDNA during reverse transcription. Unlike HIV, SFV primarily undergoes reverse transcription prior to infection of new cells, and only the DNA already present in the virion gets incorporated into new cells [39], [40]. Thus, evidence of human APOBEC activity acting on SFV implies at least one round of replication within the human host. This study provides the first evidence, although indirect, supporting SFV replication in humans. However, this conclusion is in contrast to other work failing to detect SFV replication in human oral or blood cells using other methods [7]. Indeed, in a companion study [9] we were unable to detect SFV RNA in buccal swab samples from the same seropositive humans. This suggests that the level of replication in humans may be below the limit of detection, which is consistent with the overall low proviral titers observed in human blood.

Almost half of the human host SFV *gag* sequences in this study contained in-frame stop codons within the coding region, which would prevent further replication. Although there are likely to be replication competent proviruses in humans, our studies have failed to detect any SFV transcripts. We cannot say there are no transcripts, only that our RT-PCR methods have failed to detect these.

We also could not exclude the possibility that there is a strain- or host-level effect on hypermutation frequency. In Feeroz et al. [3] we demonstrated that SFV *gag* sequences from free-ranging rhesus macaques in Bangladesh primarily cluster into six strains, and that these strains have a strong correspondence with sampling location and/or origin of the animal. Here we observe that some of these SFV strains show more evidence of hypermutation than others (Table 2). Two humans and 10 monkeys were infected with the *karamjal* strain, a strain characteristically found in animals that originate from the Karamjal region of Bangladesh. Only one out of the 73 sequences of the *karamjal* strain was found to be hypermutated, and that one hypermutated sequence was from a macaque. Additionally, no hypermutated sequences were found in a human infected with the *charmaguria* strain, a strain detected in the macaques in the town of Charmaguria. On the other hand, 22 of the 31 sequences in *bormi2* sequenced from human hosts (see [25] for terminology) were positive for hypermutation, and every human of the four infected with *bormi2* had at least one hypermutated sequence. This contrasts with only one sequence of the 102 *bormi2* sequences obtained from monkey hosts being positive for hypermutation. Additional data are required to understand how viral strain and host response influence hypermutation.

## Materials and Methods

### Data set

The data set is completely described in [3], [25]. The human study population consisted of eight human subjects who were found to be positive for SFV by PCR as part of a larger study, as well as 169 free-ranging macaques (*M. mulatta*). The macaques and humans were sampled in regions of Bangladesh where they come into close contact in the context of daily life. RT-PCR was performed to clone partial *gag* sequences (1125 bp) from buccal swab RNA of 30 macaques [9], while *gag* proviral sequences were PCR amplified and sequenced from blood of macaques and humans. An average of six clones per sample were sequenced.

### Computational analysis

#### Clustering methods

Both strain classification and the hypermutation analysis methods described below require robust clustering methods. It was found that, particularly with higher clustering thresholds, UCLUST v1.1 [41] produced poor clustering results due to the greedy nature of the algorithm. An iterative recentering algorithm, suggested by the UCLUST author at http://drive5.com/usearch/manual/recenter.html, was implemented which helped with this issue. For each round of clustering, consensus sequences from the prior round were added to the top of an ungapped alignment, in order of cluster size from greatest to least; during that iteration, clustering was carried out using cluster_smallmem, producing new consensus sequences and clusters. This process was repeated twice. Cluster assignments were further fine-tuned by a script that found the true centroid of each cluster, as defined by the sequence with minimal average normalized Hamming distance to every other sequence in the cluster. Each sequence was checked by this script to make sure that it clustered with the centroid to which it was closest (again, as defined by normalized Hamming distance), and reassignments were made as necessary.

#### Characterization of hypermutation

Methods of hypermutation evaluation typically compare sequences of interest to some putatively non-hypermutated sequence, which we refer to as the reference sequence. The authors of the HYPERMUT tool suggest using subtypes. Because subtypes are not defined for SFV we used an iterative clustering algorithm to obtain appropriate reference sequences and minimize the effect of phylogenetic signal on these comparisons (Figure S5).

During the first iteration, each sequence was compared to a consensus of all of the sequences. As described below, sequences were flagged as hypermutation positive or negative relative to several hypermutation patterns. For sequences marked as positive in any of these patterns, the sites marked as of hypermutated were removed from this global alignment, producing a draft hypermutation negative alignment.

The algorithm then proceeds iteratively from this starting point. For each successive iteration, sequences from the previous iteration's draft hypermutation-negative alignment were run through a clustering algorithm. The original sequences were then compared to the consensus sequence of its cluster as a reference sequence. The first step in clustering was to run the iterative recentering clustering algorithm described above at a 98.8% identity. To avoid potential issues induced by small clusters composed mostly of hypermutated sequences being compared to their consensus sequences which reflect this hypermutation, clusters with fewer than 15 sequences were merged with the closest cluster, as defined by the distance between cluster centroids, until no clusters smaller than 15 sequences remained. Distances were computed using the ape package's dist.dna [42] under the K80 [43] model.

The 98.8% clustering threshold for obtaining reference sequences was chosen to partition sequences based on sub-strain phylogenetic structure. Lower thresholds led to false positive identification of hypermutation in entire small clades. These clades were separated from the *dhamrai* strain by phylogenetic signal derived in part by a number of GG to GA mutations. These mutations occurred in similar locations in the sequences obtained from several distinct specimens with their correspondingly different sequences. We did not observe such positional hypermutation similarity across multiple sequences in the rest of the data, nor did we see entire clades being marked as hypermutation positive. This suggested that the mutations defining these clades occurred in the common ancestor of the sequences rather than being the result of recent hypermutation. At 98.8% we did not observe this phenomenon.

The entire process of hypermutation evaluation and clustering was repeated for 5 iterations. Hypermutation results from the final iteration were taken as the definitive results for the data set.

#### Bayesian method of hypermutation quantification

The statistical question underlying hypermutation detection is formulated as follows. Assume we have two different contexts *A* and *B*, and some number of trials is performed in each context; each trial has some probability of success. We are interested in comparing the probability of success in context *A* to that in *B*. In our application, we consider contexts as nucleotide contexts, trials as the number of such contexts, and successes as G to A mutations in a context.

Here we apply Bayesian perspective to estimate the posterior distribution of the relative probability of mutations in two nucleotide contexts of interest. We will do so by considering the mutation probability in each of the two nucleotide contexts as random variables and taking their ratio to get the relative probability ratio. In the Bayesian framework, one starts with a so-called *prior distribution* on the model parameter representing a vague posterior in the absence of data. Then using this prior and data, one is able to obtain an informed distribution estimate of the model parameter called the *posterior distribution*. In our setting, because the priors on the individual mutation probabilities are identical, the resulting prior for the ratio places equal prior probability on the relative probability being greater than one and less than one.

We consider the number of mutations in each context to be independent binomially-distributed random variables where *r* and *s* are the number of successes and failures, respectively:
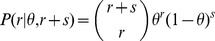
We model the probability of success for the binomial random variable with a beta distribution. The beta distribution is a classical distribution *Beta* on [0,1] parameterized by *a* and *b* with density
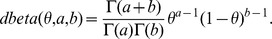
For example, *Beta*(1,1) is the uniform distribution. If the prior distribution is *Beta*(*a,b*), then given the observation of *r* successes and *s* failures under a binomial model, the posterior distribution is 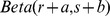 [44].

The probability distribution function (PDF) for the ratio *w* of two beta-distributed random variables (denoted *θ* above) was found in 2000 by Pham-Gia [45] in terms of hypergeometric and beta functions. The PDF uses the hypergeometric function
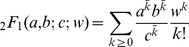
where 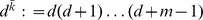 is the *k*th rising power of a.

Theorem (Pham-Gia, 2000): Assume that 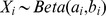 for 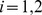. Then the density of 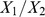 for 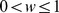 is
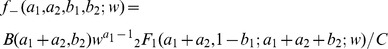
and for 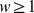 is
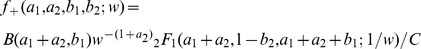
where 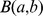 is the beta function, _2_ *F*_1_ is the hypergeometric function, and 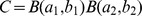.

We will denote the probability distribution corresponding to this density as *BetaRat*(*a*_1_,*a*_2_,*b*_1_,*b*_2_).

If we run two experiments 1 and 2 with 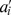 successes and 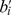 failures for experiment *i*, then the posterior on the ratio of 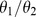 will be that of the above theorem with 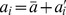 and 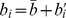, assuming a prior distribution of 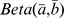 for each experiment. In summary, with count priors 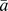 and 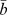, and 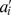 successes and 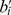 failures for experiment *i*, the relative probability ratio will have the distribution
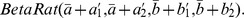
As one might expect, there is a symmetry in the theorem when the subscripts 1 and 2 are exchanged and *w* inverted. Specifically,
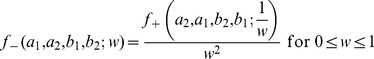
and
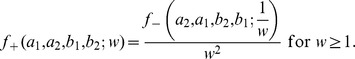
It turns out for some parameter regimes the calculation of 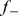 and 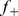 is especially difficult, and we can use these equations to move into a different regime.

Care is required in evaluating _2_ *F*_1_ in the regime of interest here. Specifically, we are interested in evaluating 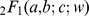 in the case where *a*, *b*, and *c* can be in the hundreds in magnitude, and *w* is near one. Using the implementation of the hypergeometric function in the GNU Scientific Library, Mathematica, scipy or R resulted in numerical instability for this domain. In fact, a direct summation using the above definition leads to problems when *w* is close to or greater than one because the rising factorial powers and the factorial can compensate for each other:
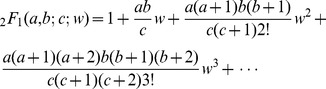
When this is the case, the convergence of the series depends on the powers of *w*.

Others in the applied mathematics community have encountered difficulties calculating hypergeometric functions when *w* is close to 1; for example [46] proposes a complex system of equations for evaluating _2_ *F*_1_ when *w* is close to 1 and other challenging situations. However, even this does not consistently result in numerically stable solutions when *a* and *b* are relatively large without employing high/multiple precision arithmetic, as used in the python mpmath library's implementation of the function.

In the special case of only moderately negative, integral values of *b* (recall that *b* is either 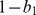 or 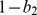 in our setting), the case for the majority of our domain, we profit from applying the recursively factored form
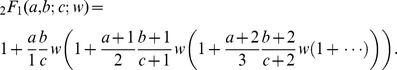
For such values of *b*, this potentially infinite product is in fact finite. When the counts are moderately large, direct evaluation is feasible. A simple C language implementation of this product employing the GMP library is approximately an order of magnitude faster than mpmath in cases of moderate magnitudes of *b* (on the order of a couple hundred). As the magnitude of *b* increases, the number of iterations needed to compute the recursively factored form becomes large and this approach becomes less efficient. We have the option of switching to the mpmath implementation in these cases.

The prior chosen for these analyses is based on a *Beta*(0.5, 1.0) distribution, which has an expectation of 1/3. This distribution represents our prior belief that mutations are generally rare within any given context. This Beta distribution translates to a relatively uninformative *BetaRat*(0.5, 0.5, 1.0, 1.0) prior of the Beta ratio distribution. We note that this prior does not bias us towards either the focus or control context, and was found to push the ratio towards 1 in the case of sparse information better than a *BetaRat*(1.0, 1.0, 1.0, 1.0) prior.

Using these implementations we can apply direct numerical quadrature to get the MAP value and 95% confidence interval of the posterior.

#### Hypermutation quantification with Fisher's test and the risk ratio

Here we review our use of these classical methods for clarity. Given *a_i_* G sites mutated to A and *b_i_* G sites not mutated to A for context *i* = 1,2(corresponding to in-context and out of context respectively in our setting), we apply the Fisher exact test to the contingency table
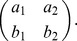
These tests were computed using the fisher python library [https://pypi.python.org/pypi/fisher/]. The mid-P variant of this test was computed using a form of the ormidp.test function from the R epitools package [http://cran.r-project.org/web/packages/epitools/index.html] modified to allow for computation of P-values for a strict one-tailed alternative hypothesis.

The basic formula for the risk ratio in this setting is
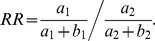
To avoid the case where the denominator is zero, we can add pseudocounts of 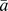 and 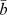 to obtain
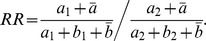
(We note that the output of the HYPERMUT website shows an equation that is labeled “rate ratio” but appears to be the formula for the odds ratio. By trying example sequences it becomes clear that they are computing the risk ratio.)

#### Hypermutation evaluation: Implementation

Note that the context for a given G to A mutation is defined to be the context on the potentially hypermutated sequence, not the context on the reference sequence; this is also the default setting with HYPERMUT. These context patterns will be referred to by their focus context in terms of degenerate IUPAC codes. For brevity, hA3*X* denotes a human APOBEC3*X* enzyme, while rhA3*X* denotes a rhesus APOBEC3*X* enzyme.

The GG, GA and GR patterns correspond to the hypermutation patterns used in HYPERMUT [19] to evaluate for presence of hypermutation associated with primarily APOBEC3G activity, primarily APOBEC3F activity and combined A3G/A3F activity (respectively). It was found that several of the most hypermutated sequences from macaques bore a high number of mutations not only in a GA context, but also in a GC context. This context matches that observed in rhesus macaque A3DE (rhA3DE) hypermutation, as ascertained by transfection studies [29]. In order to more effectively detect this type of activity, we evaluated sequences for the GM mutation pattern. Additionally, in order to detect hypermutation resulting from combined rhA3F (or possibly other GA-context rhA3) and rhA3DE activity, we added the GH (G followed by anything but a G) pattern.

We called the sequence as hypermutated in a given context when the corresponding Q05 (the 0.05 quantile as described above) value of the posterior distribution for the probability ratio exceeded 1. For each sequence identified as hypermutated in more than one context, the context with the highest Q05 value was identified as the call pattern. The call pattern therefore represents the context in which evidence of hypermutation is strongest.

The hypermutation analysis was carried out using our implementation of the above method, called *hyperfreq*, which is publicly accessible at http://github.com/fhcrc/hyperfreq. The core component responsible for evaluation of the *BetaRat* distributions is available as a separate module at http://github.com/fhcrc/betarat.

#### Simulation validation

For the first validation, mutation counts were simulated from an array of control context mutation probabilities and relative probability ratios in a parameter regime mimicking that observed for the SFV sequences. For each control context probability and relative probability ratio pair, mutation counts for the control and focus contexts were generated by sampling from the corresponding binomial distributions. The number of focus and control context positions – 75 and 225, respectively – were chosen to approximate the number of GG vs GH (G followed by something other than a G) positions we would expect from a SFV *gag* sequence of length 1200. To mimic the HIV data that was used for real-data validation (see below), we also simulated sequences of length 600. Additional sequence lengths were simulated for supplementary figures. 1000 count-based simulations were done in this manner for each parameter set to compare hypermutation detection methods, and 5000 were done for the effect size comparison. From these counts, MAP estimates were computed and compared to the corresponding relative probability ratio used for simulation, as were RRs. Pseudocounts were added to the observations to avoid division by zero for the RR. For fairness, we employed pseudocounts corresponding to the prior used in the Bayesian analysis: given mutation counts a and b in the focus and control contexts, respectively, the RR was computed as ((a+0.5)/76)/((b+0.5)/226) for the 1200 bp simulations and ((a+0.5)/39)/((b+0.5)/113) for the 600 bp simulations. The mean squared error (MSE) was computed for each estimator and parameter set, and used to calculate the ratio of the MSE for the RR and mid-P to that of the MAP estimator (Figure 2). The ROC curves (Figure S4) were generated by a custom R script and aggregated across various simulation parameters (control or background mutation probability, sequence length, and true RPR).

For the second validation of our hypermutation detection framework, we developed a simulation framework using the bppsuite programs [47]. First, a phylogenetic tree from the [27] sequences was built using FastTree [48] and a maximum likelihood mutation matrix was derived for the corresponding alignment using the Bio++ library [49] under the HKY85 model [50], yielding model parameters kappa = 56.74, theta = 0.3145, theta1 = 0.7385, theta2 = 0.5263. Sequences were simulated using that mutation matrix and nucleotide distribution, creating a simulated data set with compositional and mutational similarity to the Refsland *et al.* [27] data. However, because these sequences were made from a random process that simulates each nucleotide position independently, there is no dinucleotide specificity and so any detection of hypermutation is a false positive.

#### Real data validation

For the Refsland data, we had the original, unmutated sequences so we didn't have to do clustering and unmutated sequence inference. Otherwise the analysis was the same.

To process the Land et al. [28] data, we used HIV subtype reference sequences for comparison in order to find hypermutation. Specifically, for each sequence evaluated, we took the best BLAST hit of that sequence on the LANL subtype reference sequences from 2010 (in HXB2 range 5979–6576; http://www.hiv.lanl.gov/content/sequence/NEWALIGN/align.html) and used that as the un-mutated ancestor. We chose this fully-automated approach rather than a similar but semi-automated approach used in the original paper, which required expert knowledge. BLAST hits and query sequences were aligned together using MUSCLE v3.8.31 [51] and trimmed with trimal v1.3.rev14 [52] using the –gappyout setting, and the resulting sequences were used as input to hyperfreq. As in the original study, only the single directly sequenced PCR product for each patient was analyzed with respect to that individual's CD4 count. For the risk ratio computations we used (1, 1) pseudocounts.

## Supporting Information

Figure S1 A simple example showing how the ratio of MAP values for two Beta distributions is not the same as the MAP value of the corresponding BetaRat distribution. The MAP ratio calculated is MAP(*Beta(2.0, x)*)/MAP(*Beta(x, 2.0)*), while the BetaRat MAP calculated is MAP(*BetaRat(2.0, x, x, 2.0)*).
(TIFF)

Figure S2 Comparison of P-value cumulative density functions (CDFs) under the null for the Fisher exact test, mid-P, and BetaRat methods. Individual plots are faceted by sequence length (rows) and control context mutation probability (columns). In the frequentist paradigm, p-values should be uniformly distributed on the unit interval under the null hypothesis, corresponding to the y = x line for the CDF. The classical Fisher P-value is consistently conservative, while the mid-P and BetaRat CDFs are much closer to what would be expected under the null, especially for the range<0.05.
(TIFF)

Figure S3 Comparison of P-value (PPF for BetaRat) cumulative density under the various true RPRs (rows) for Fisher exact test, mid-P, and BetaRat methods. Plot columns correspond to increasing control context mutation probabilities. Both the mid-P and BetaRat methods are consistently more powerful than the Fisher exact test, with P-value distributions closer to the null, as also supported in Figure S2.
(TIFF)

Figure S4 Aggregated Receiver Operating Characteristic (ROC) curves for three methods of assessing significance under simulation. Numbers in the plot show the actual relative probability ratio used for simulation. These curves show the trade-off between sensitivity and specificity, in that a point represents the true positive rate that can be achieved given a certain level of false positive rate by adjusting the cutoff. Note that these curves say nothing about selecting these cut-offs, which is addressed in the other plots. Our formulation “br_cdf” has the highest line in each category, and thus has the best such tradeoff.
(TIFF)

Figure S5 Flow of data throughout the analysis, from the original alignment, through iterative hypermutation analysis, strain clustering and other downstream analyses.
(TIFF)

Figure S6 Highlighter (http://www.hiv.lanl.gov/content/sequence/HIGHLIGHT/highlighter.html) plot showing mutations in a number of bormi2 SFV sequences obtained from both monkeys and humans. The labeling of mutations as being APOBEC-associated or not was made by the Highlighter tool and may or may not correspond to what we find with our methodology.
(TIFF)

Table S1 Our methodology (denoted Q05) is more sensitive than the Fisher test on the Refsland data set and does not increase the false positive rate. GG and GA were used as focus context for the tests on data from both normal cells along with A3F (GA context) and A3G (GG context) knockouts. The numerical entries show the percent of viral sequences called hypermutated. Here A3 is used as an abbreviation for APOBEC3.
(DOCX)

Table S2 Statistics on the percentage of sequences called hypermutated by Q05 on data sets simulated without hypermutation from the Refsland sequences (see Materials and Methods). All entries of the equivalent table for the Fisher test at a 5% significance level were zero. Thus the median positive probability for Q05 is closer to 5% than for Fisher, although it was still conservative for this data set.
(DOCX)
