# Supplementary figures and images for: A Novel Bayesian Method for Detection of APOBEC3-Mediated Hypermutation and Its Application to Zoonotic Transmission of Simian Foamy Viruses
Source: PLoS Comput Biol. 2014 Feb 27;10(2):e1003493. doi: 10.1371/journal.pcbi.1003493 (PMC3937129; doi:10.1371/journal.pcbi.1003493)

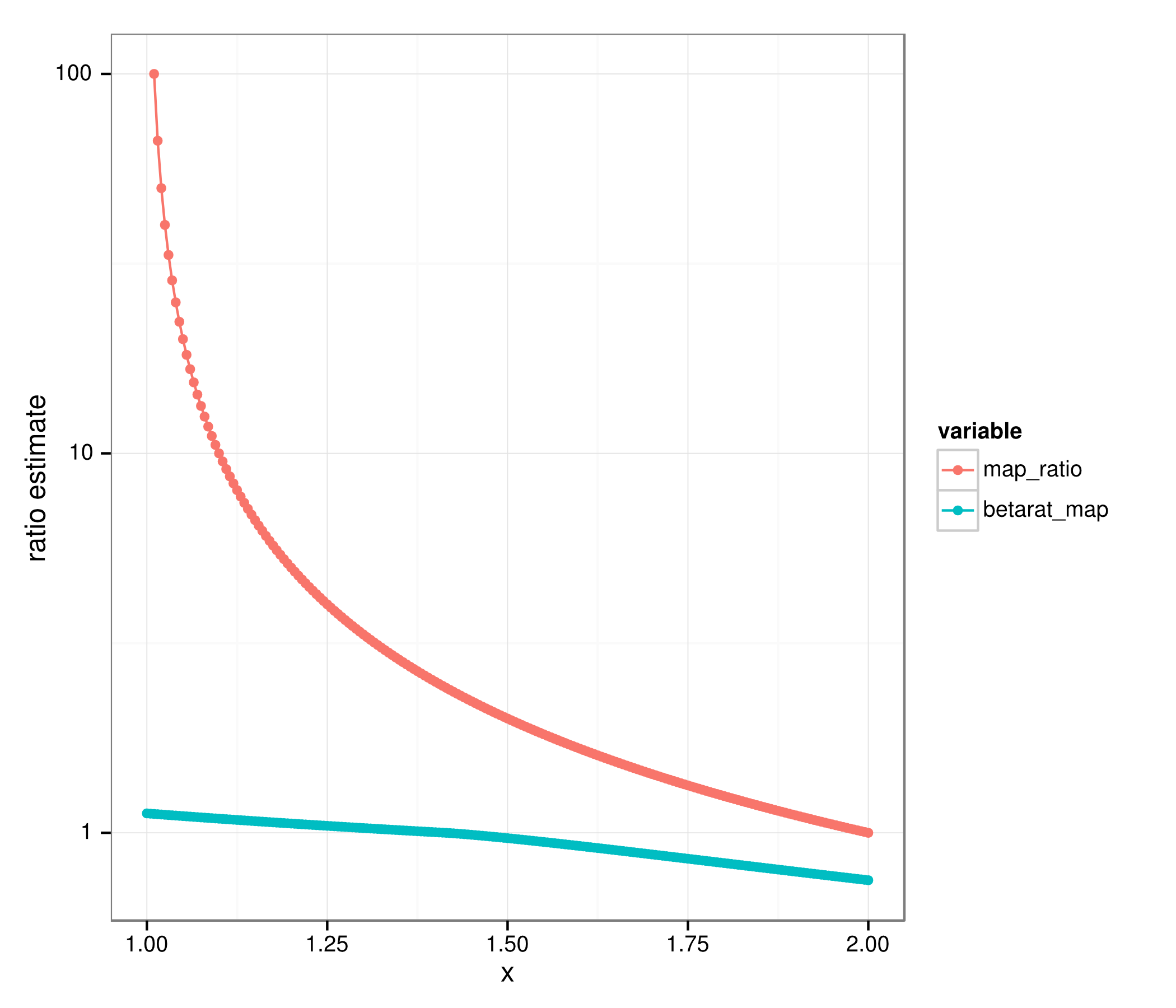

Supplement: Figure S1 — A simple example showing how the ratio of MAP values for two Beta distributions is not the same as the MAP value of the corresponding BetaRat distribution. The MAP ratio calculated is MAP(Beta(2.0, x))/MAP(Beta(x, 2.0)), while the BetaRat MAP calculated is MAP(BetaRat(2.0, x, x, 2.0)). (TIFF) [file pcbi.1003493.s001.tif]

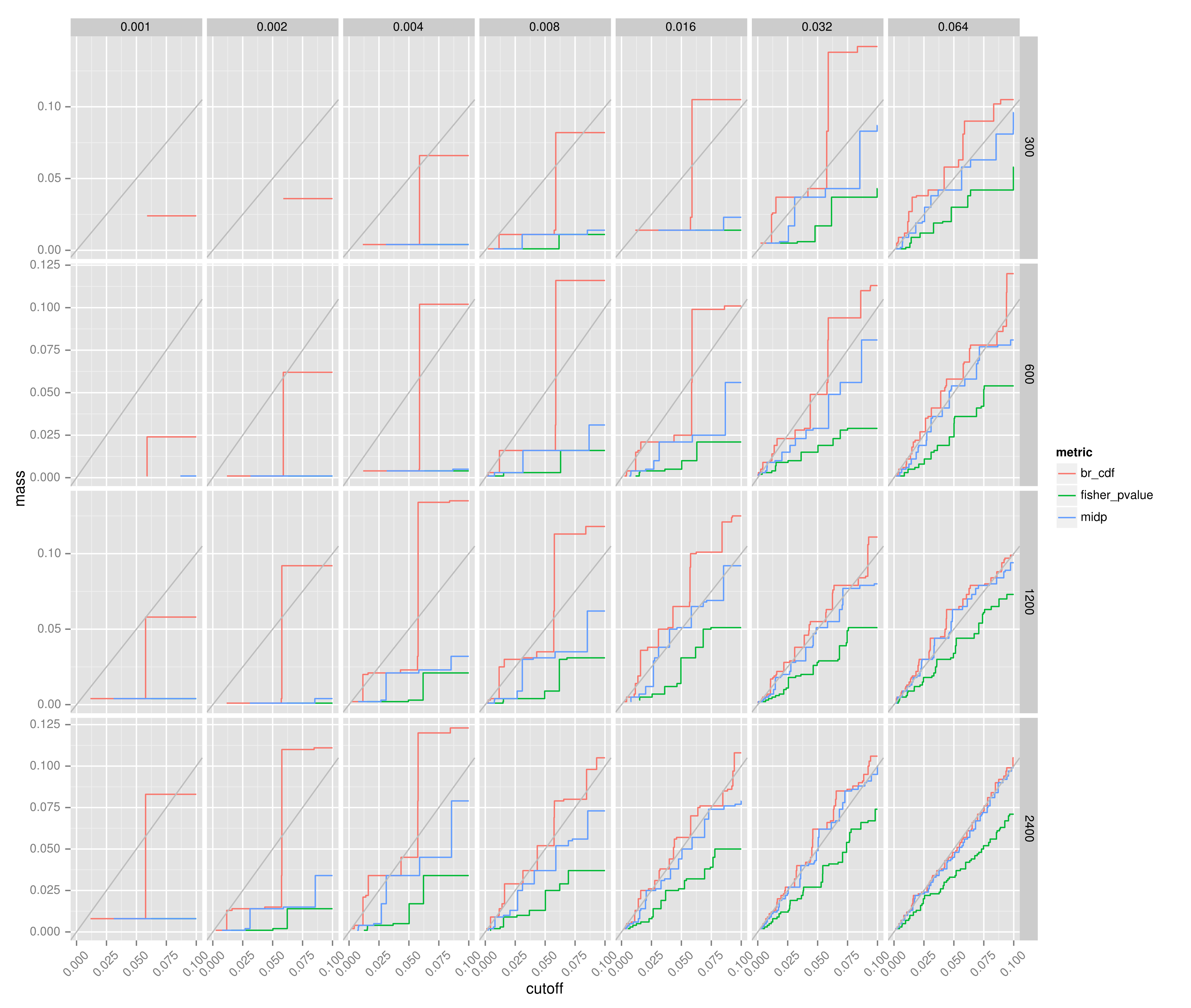

Supplement: Figure S2 — Comparison of P-value cumulative density functions (CDFs) under the null for the Fisher exact test, mid-P, and BetaRat methods. Individual plots are faceted by sequence length (rows) and control context mutation probability (columns). In the frequentist paradigm, p-values should be uniformly distributed on the unit interval under the null hypothesis, corresponding to the y = x line for the CDF. The classical Fisher P-value is consistently conservative, while the mid-P and BetaRat CDFs are much closer to what would be expected under the null, especially for the range<0.05. (TIFF) [file pcbi.1003493.s002.tif]

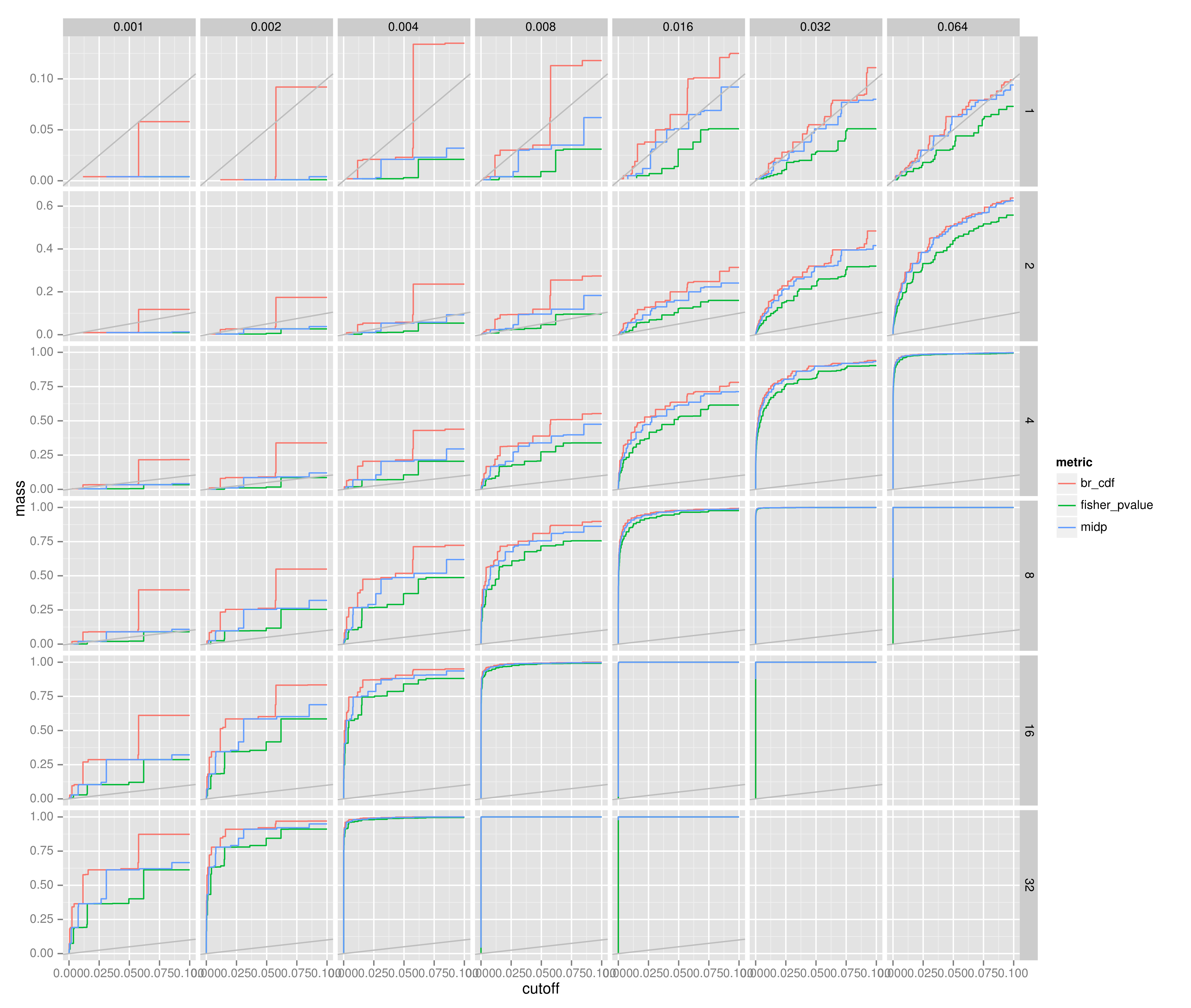

Supplement: Figure S3 — Comparison of P-value (PPF for BetaRat) cumulative density under the various true RPRs (rows) for Fisher exact test, mid-P, and BetaRat methods. Plot columns correspond to increasing control context mutation probabilities. Both the mid-P and BetaRat methods are consistently more powerful than the Fisher exact test, with P-value distributions closer to the null, as also supported in Figure S2. (TIFF) [file pcbi.1003493.s003.tif]

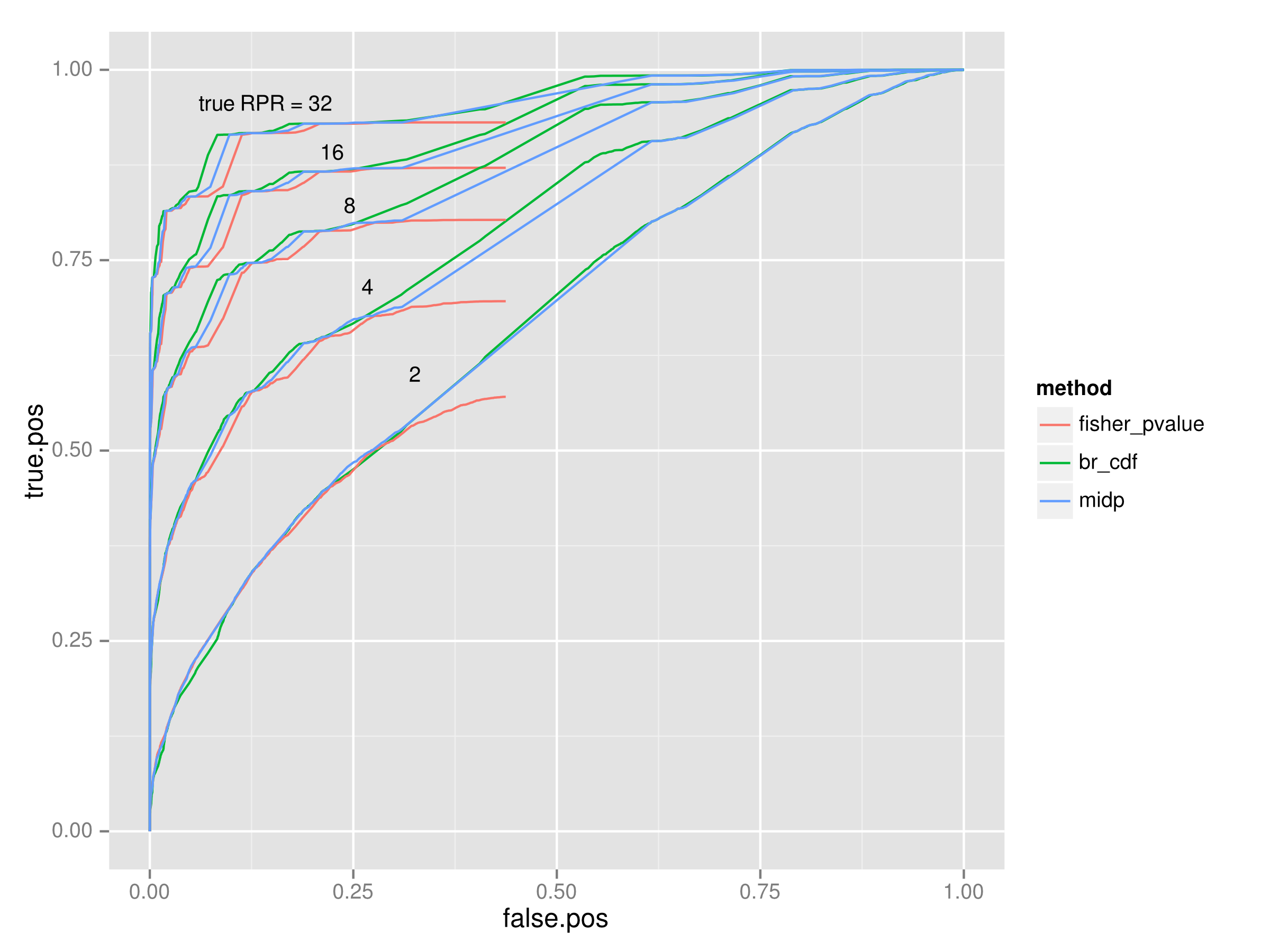

Supplement: Figure S4 — Aggregated Receiver Operating Characteristic (ROC) curves for three methods of assessing significance under simulation. Numbers in the plot show the actual relative probability ratio used for simulation. These curves show the trade-off between sensitivity and specificity, in that a point represents the true positive rate that can be achieved given a certain level of false positive rate by adjusting the cutoff. Note that these curves say nothing about selecting these cut-offs, which is addressed in the other plots. Our formulation “br_cdf” has the highest line in each category, and thus has the best such tradeoff. (TIFF) [file pcbi.1003493.s004.tif]

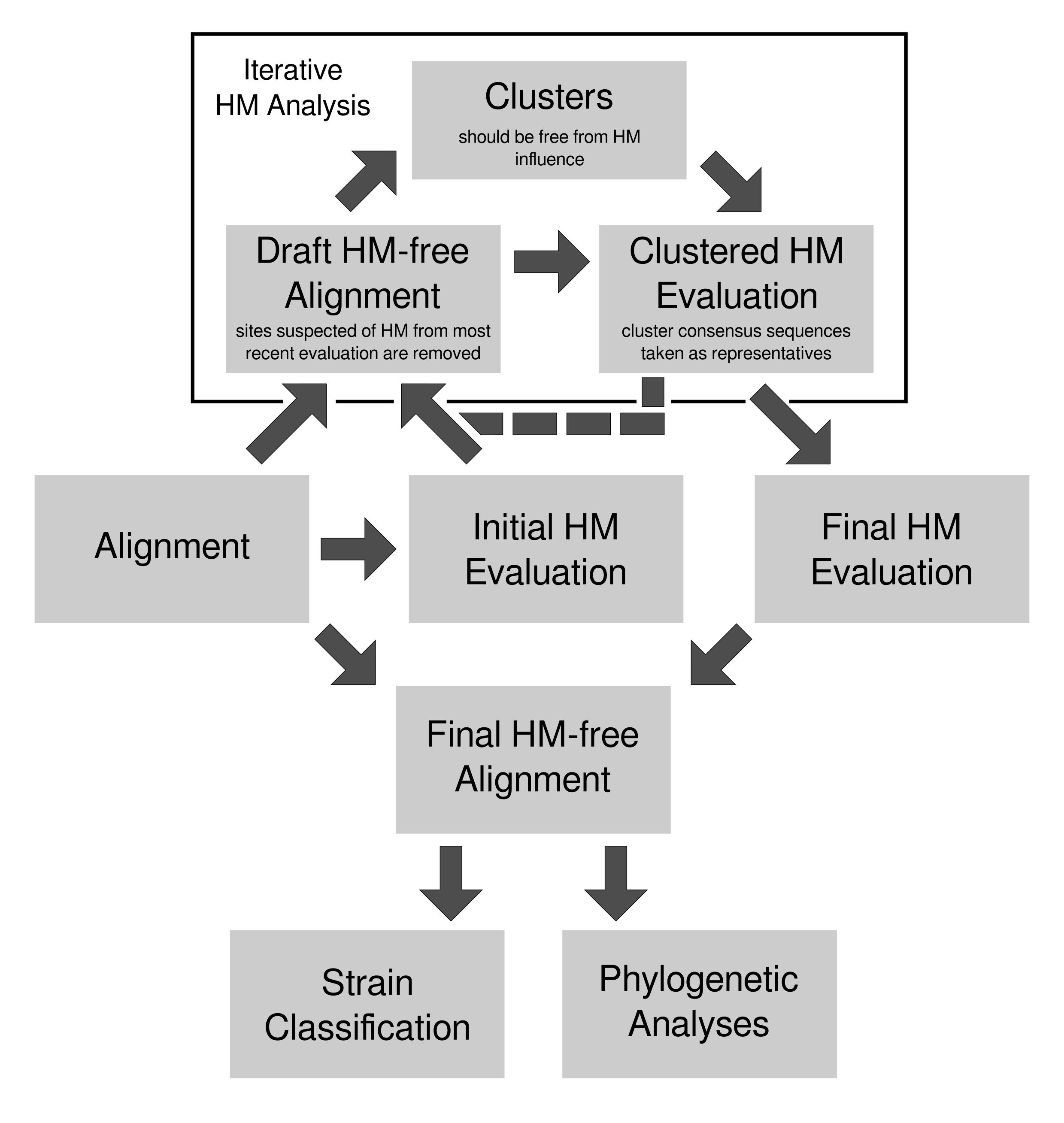

Supplement: Figure S5 — Flow of data throughout the analysis, from the original alignment, through iterative hypermutation analysis, strain clustering and other downstream analyses. (TIFF) [file pcbi.1003493.s005.tif]

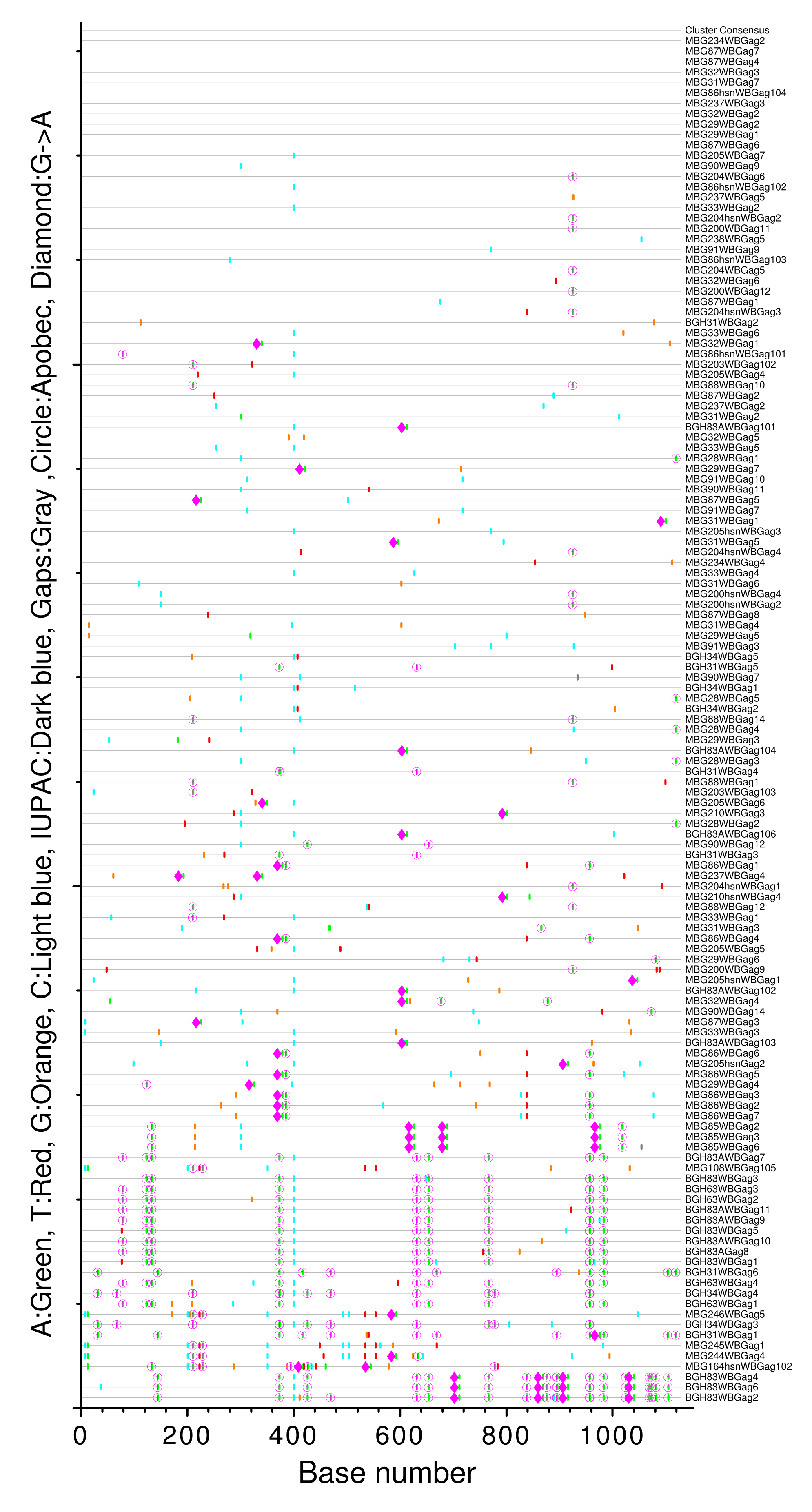

Supplement: Figure S6 — Highlighter (http://www.hiv.lanl.gov/content/sequence/HIGHLIGHT/highlighter.html) plot showing mutations in a number of bormi2 SFV sequences obtained from both monkeys and humans. The labeling of mutations as being APOBEC-associated or not was made by the Highlighter tool and may or may not correspond to what we find with our methodology. (TIFF) [file pcbi.1003493.s006.tif]
