# Supplementary material for: A Novel Bayesian Method for Detection of APOBEC3-Mediated Hypermutation and Its Application to Zoonotic Transmission of Simian Foamy Viruses
Source: PLoS Comput Biol. 2014 Feb 27;10(2):e1003493. doi: 10.1371/journal.pcbi.1003493 (PMC3937129; doi:10.1371/journal.pcbi.1003493)
Supplement: Table S1 — Our methodology (denoted Q05) is more sensitive than the Fisher test on the Refsland data set and does not increase the false positive rate. GG and GA were used as focus context for the tests on data from both normal cells along with A3F (GA context) and A3G (GG context) knockouts. The numerical entries show the percent of viral sequences called hypermutated. Here A3 is used as an abbreviation for APOBEC3. (DOCX) [file pcbi.1003493.s007.docx]

| focus context | data set | % positive by Q05 | % positive by Fisher |
| --- | --- | --- | --- |
| GG | ΔA3F | 10 | 10 |
| GG | ΔA3G | 0 | 0 |
| GG | normal | 11.67 | 10 |
| GA | ΔA3F | 3.33 | 3.33 |
| GA | ΔA3G | 26.67 | 10 |
| GA | normal | 8.33 | 5 |
