# Supplementary material for: A Novel Bayesian Method for Detection of APOBEC3-Mediated Hypermutation and Its Application to Zoonotic Transmission of Simian Foamy Viruses
Source: PLoS Comput Biol. 2014 Feb 27;10(2):e1003493. doi: 10.1371/journal.pcbi.1003493 (PMC3937129; doi:10.1371/journal.pcbi.1003493)
Supplement: Table S2 — Statistics on the percentage of sequences called hypermutated by Q05 on data sets simulated without hypermutation from the Refsland sequences (see Materials and Methods). All entries of the equivalent table for the Fisher test at a 5% significance level were zero. Thus the median positive probability for Q05 is closer to 5% than for Fisher, although it was still conservative for this data set. (DOCX) [file pcbi.1003493.s008.docx]

| Pattern | data_set | 1st quartile | Median | 3rd quartile |
| --- | --- | --- | --- | --- |
| GG | ΔA3F | 0 | 0 | 0 |
| GG | ΔA3G | 0.833 | 3.33 | 9.17 |
| GG | original | 0 | 1.67 | 4.58 |
| GA | ΔA3F | 0 | 0 | 0 |
| GA | ΔA3G | 0 | 0 | 0 |
| GA | original | 0 | 0 | 1.67 |
